# Training models and simulators for endoscopic transsphenoidal surgery: a systematic review

**DOI:** 10.1007/s10143-023-02149-3

**Published:** 2023-09-19

**Authors:** Giacomo Santona, Alba Madoglio, Davide Mattavelli, Mario Rigante, Marco Ferrari, Liverana Lauretti, Pierpaolo Mattogno, Claudio Parrilla, Pasquale De Bonis, Jacopo Galli, Alessandro Olivi, Marco Maria Fontanella, Antonio Fiorentino, Mauro Serpelloni, Francesco Doglietto

**Affiliations:** 1 Department of Information Engineering, University of Brescia, Brescia, Italy; 2 Department of Morphology, Surgery and Experimental Medicine, University of Ferrara, Ferrara, Italy; 3 Department of Neurosurgery, Sant’ Anna University Hospital, Ferrara, Italy; 4 Otorhinolaryngology-Head and Neck Surgery, Department of Medical and Surgical Specialties, Radiological Sciences, and Public Health, ASST Spedali Civili of Brescia, University of Brescia, Brescia, Italy; 5 Otorhinolaryngology, Fondazione Policlinico Universitario A. Gemelli IRCCS, Rome, Italy; 6 Section of Otorhinolaryngology-Head and Neck Surgery, Department of Neurosciences, University of Padua - Azienda Ospedaliera di Padova, Padua, Italy; 7 Neurosurgery, Department of Neurosciences, Sensory Organs and Thorax, Università Cattolica del Sacro Cuore, Rome, Italy; 8 Neurosurgery, Fondazione Policlinico Universitario A. Gemelli IRCCS, Rome, Italy; 9 Otorhinolaryngology, Department of Neurosciences, Sensory Organs and Thorax, Università Cattolica del Sacro Cuore, Largo Agostino Gemelli, 8, 00168 Rome, Italy; 10 Neurosurgery, Department of Medical and Surgical Specialties, Radiologic Sciences, and Public Health, University of Brescia - ASST Spedali Civili di Brescia, Brescia, Italy; 11 Department of Mechanical and Industrial Engineering, University of Brescia, Brescia, Italy

**Keywords:** 3D printing, Arachnoid, Pituitary adenoma, Cadaveric head, Animal head, Training models, Training simulators, Transsphenoidal surgery

## Abstract

Endoscopic transsphenoidal surgery is a novel surgical technique requiring specific training. Different models and simulators have been recently suggested for it, but no systematic review is available. To provide a systematic and critical literature review and up-to-date description of the training models or simulators dedicated to endoscopic transsphenoidal surgery. A search was performed on PubMed and Scopus databases for articles published until February 2023; Google was also searched to document commercially available. For each model, the following features were recorded: training performed, tumor/arachnoid reproduction, assessment and validation, and cost. Of the 1199 retrieved articles, 101 were included in the final analysis. The described models can be subdivided into 5 major categories: (1) enhanced cadaveric heads; (2) animal models; (3) training artificial solutions, with increasing complexity (from “box-trainers” to multi-material, ct-based models); (4) training simulators, based on virtual or augmented reality; (5) Pre-operative planning models and simulators. Each available training model has specific advantages and limitations. Costs are high for cadaver-based solutions and vary significantly for the other solutions. Cheaper solutions seem useful only for the first stages of training. Most models do not provide a simulation of the sellar tumor, and a realistic simulation of the suprasellar arachnoid. Most artificial models do not provide a realistic and cost-efficient simulation of the most delicate and relatively common phase of surgery, i.e., tumor removal with arachnoid preservation; current research should optimize this to train future neurosurgical generations efficiently and safely.

## Introduction

Endoscopic transsphenoidal surgery is a novel surgical technique that recently evolved in endoscopic skull base surgery [1]. As we have learned to exploit the advantages of the relatively large median and paramedian corridors to the skull base [2–5], the indications for this surgery have been expanding, together with its complexity.

It is well recognized that endoscopic transsphenoidal surgery has a long learning curve [6], which requires integrated and specific training [7]. Though traditional neurosurgical training is still primarily based on experience in the operating room, many complementary methods are now available. The cadaver laboratory has been classically used to acquire basic technical skills and knowledge of detailed surgical anatomy. Still, high maintenance costs and the challenge of simulating pathologies might limit its utility. Thanks to 3D printing technologies, it has become possible to create customized models replicating normal and pathological anatomy [8]. Furthermore, thanks to virtual reality (VR) development, simulators may provide a repeatable experience in a more complex anatomical environment. In addition, the development of augmented reality (AR) simulators might enhance the quality of training.

In this evolving scenario, this review aims to provide a systematic and up-to-date description of the training solutions for endoscopic transsphenoidal surgery, along with their technical details, costs, utility for surgical skills development, and validation.

## Material and methods

### Search strategy

A systematic review, following the PRISMA 2020 statement [9, 10], was performed by searching articles published until February 2023 on PubMed and Scopus, with the following keywords: training AND (transsphenoidal OR transnasal) AND (phantom OR simulator OR model); physical AND (simulator OR phantom OR model) AND (endoscopic endonasal); (Pituitary OR hypophysis) AND surgery AND training AND (model OR phantom OR simulator); (transsphenoidal) AND ((3D print*) OR (three dimension* print*)); ((3D print*) OR (three dimension* print*)) AND tumor AND pituitary OR hypophysis; (Endoscopic endonasal) AND ((3D print*) OR (three dimension* print*)); (neurosurgical) AND training AND ((phantom) OR (model) OR (simulator)) AND (3D print*) OR (three dimension* print*); (Skull base) AND (surgery) AND (training) AND ((model) OR (phantom) OR (simulator)).

Additional references and models or simulators used for training in endoscopic transsphenoidal surgery were identified by reference analysis and investigations on the web using the Google search engine.

#### Inclusion and exclusion criteria

Inclusion criteria were as follows: English Language, training models, or simulators for endoscopic transsphenoidal surgery.

Exclusion criteria were the following: non-English language, papers unavailable at our libraries, models/simulators for other surgical interventions, and other studies (e.g., reviews with no novel data).

#### Quality assessment and data extraction

Articles were imported into the reference management software Zotero (version 6.0.8), and duplicates were removed. AM and GS examined the title and abstract of the retrieved records, and non-relevant citations were excluded. Any disagreement was resolved by discussion between the reviewers. For each selected study, an accurate full-text analysis was performed to extract the following information about the training model or simulator, when available: reproduced anatomy, data on training and validation studies, and costs.

**Fig. 1:**
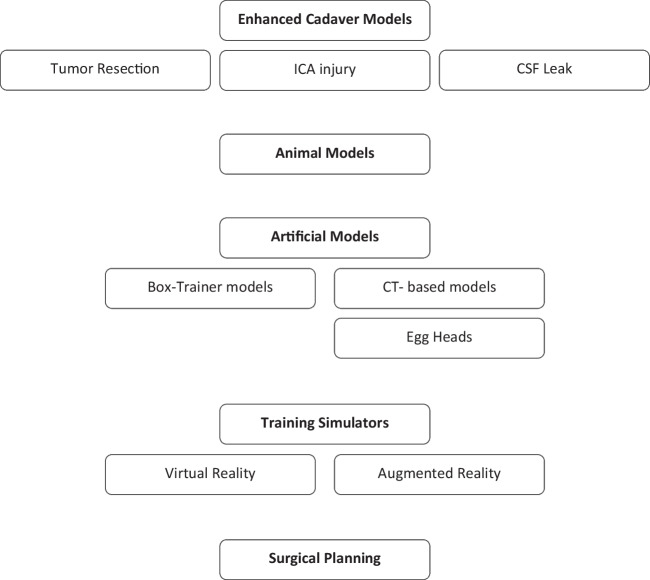
Training models and simulators diagram

The selected studies were divided into the following categories (Fig. 1):

1. Enhanced cadaver models (ECH);
2. Animal models (AM);
3. Training models;
  3.1 Box-Trainers;
  3.2 CT-based: mono-material model (m), multi-material model (M), and the “EggHead”;
4. Training Simulators: virtual reality (VR) simulator and augmented reality (AR) simulator;
5. Preoperative planning models/simulators.

The difference between “model” and “simulator” is that simulators are models in a virtual reality environment and with real-time feedback for the surgeon.

Each training model/simulator was listed in a table based on the category. In addition, each model was described in the table reporting the following data when available:

1. First author and year of publication for academic reports, or name of the developers and nation, for commercially available models/simulator (CA);
2. whether the model included the tumor (T) and the arachnoid membrane (A) in their model;
3. Simulated tasks for which the model was conceived and used;
4. Assessment or validation of the model;
5. The reported cost of the used materials or the retail price.

For the CT-based training models, a 5-point sub-column was added to evaluate their anatomical reliability and defined “anatomy score.” The sub-column score gives an overall evaluation of the anatomical accuracy of the model; points are given according to the design of the model: +1 point per mono-material (m) models or +2 points for multi-material (M) ones M; +1 or +2 points according to the degree of reproduced details, such as the skin, dura mater, optic nerve, or ICA; and +1 point if the tumor or the arachnoid are reproduced.

## Results

**Fig. 2:**
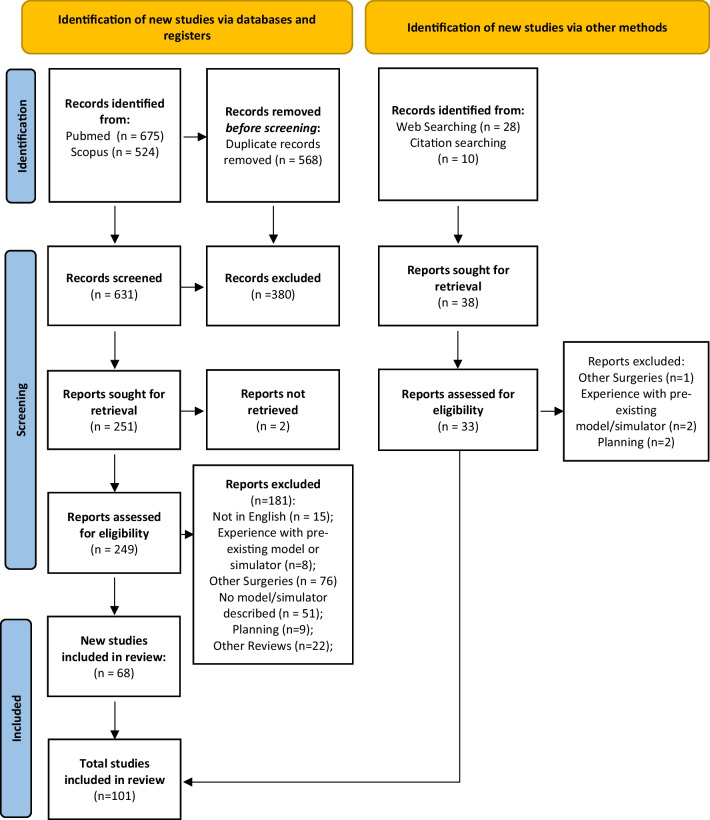
PRISMA 2020 flow chart

The initial literature search yielded 1199 articles: 675 from PubMed and 524 from Scopus. Of these articles, 568 were removed before screening because they were duplicates. The remaining 631 articles were screened and evaluated by title. At this point, 380 articles were excluded, and a full-text screening was performed to determine if the remaining 251 articles met the inclusion criteria. Of the 251 articles identified for retrieval, 2 were removed (because the full text was not available). A total of 249 reports were screened for eligibility and 181 were removed because they did not meet the inclusion criteria, specifically 15 were removed because of language; 8 were removed because of experience with a pre-existing model or simulator; 76 were removed because the model/simulator described was used to simulate other surgeries; 51 were removed because no model/simulator was described in the reports; 9 were removed because they were designed for planning; and 22 were removed because they were previous reviews of the literature. Finally, 101 reports were included in this systematic review, including 6 articles retrieved from previous papers [11–32] and 28 websites (Fig. 2).

### Enhanced cadaver models

The cadaver-based training models can be divided into three main groups, according to the training experience they provide (Table 1): tumor resection [33, 34, 38, 46], management of ICA injury [35–37, 41, 43, 44], and CSF leak repair [41, 42, 45, 47].

**Table 1:** Enhanced cadaver models. The table reports the ECH models that have been modified to simulate different surgeries, the simulated task, who assessed the model, and whenever available, the cost

FA/Dev. (YOP/nation)	ECH		Simulated task	Assessment	Cost/price
	Type	T			
Gragnaniello (2010) [33]	Tr	SRSP	SBS	Experts	n/a
Berhouma (2013) [34]	Tr	SRSP	EETA	Authors	n/a
Pham (2014) [35]	ICAI	-	ICAIR	Residents	n/a
Ciporen (2017) [36]	ICAI	-	ICAIR	Residents	600 $^a^
Pacca (2017) [37]	ICAI	-	ICAIR	Surgeons	n/a
Gagliardi (2018) [38]	Tr	SRSP	EETA	Surgeons	n/a
AlQahtani (2018, 2021) [39, 40]	CSFL	-	CSFLR	Surgeons	n/a
Shen (2018) [41]	ICAI	-	ICAI	Surgeons	n/a
Christian (2018) [42]	CSFL	-	CSFLR	Residents	n/a
Donoho (2019, 2021) [43, 44]	ICAI	-	ICAIR	Residents	275 $^b^
Mattavelli (2020) [45]	CSFL	-	CSFLR	Experts	n/a
Li (2022) [46]	Tr	-	ETTA	Surgeons	n/a

*$* US dollars, *$/head* US dollars x cadaveric head, *$/trainee* US dollars × trainee, *CSFL* cerebrospinal fluid leakage, *CSFLR* cerebrospinal fluid leakage repair, *Dev*. developers, *ECH* enhanced cadaver models, *EETA* endoscopic endonasal transsphenoidal approach, *ETTA* endoscopic transnasal transsphenoidal approach, *FA* first author, *ICAI* internal carotid artery injury, *ICAIR* internal carotid artery injury repair, *n/a* not available, *SBS* skull base surgery, *SRSP* stratathane resin ST-504 polymer, *T* tumor, *Tr* tumor resection

^a^500–700 $ per cadaver head

^b^Marginal cost per trainee

The tumor resection models are designed to train neurosurgeons to resect a sellar tumor [33, 34, 38]**.** The idea is based on the work of Gragnaniello et al. who injected resin into the sella turcica to mimic the texture and location of a pituitary tumor [33].

In ICA injury models, a red-dyed solution is pumped into the arterial system to mimic blood [35–37, 41, 43, 44]; an ICA lesion is caused, and the surgeon can be trained to deal with it.

The CSF models are obtained by perfusing a water-based solution in the subarachnoid or subdural space so that the surgeon can be trained in skull base reconstruction [39, 40, 42, 45].

Although anatomical specimens are intuitively used at their best only once, some models can be used multiple times for training, lowering their total cost [37, 41, 48]. For example, Mladina et al. [49] reported a cost of $1520 per resident.

### Animal models

These models use animals to provide training, mainly on surgical instrumentation handling (Table 2). The animals include Wistar rats [50], lambs [49, 54, 56, 58], and sheep [55, 57] and also one hybrid model specifically designed to manage ICA rupture with a live sheep [51–53].

**Table 2:** Animal-based models. The table reports animal-based training models, the simulated task, who assessed the model, and whenever available, the cost

FA/Dev. (YOP/nation)	Animal	Simulated task	Assessment	Cost/price
Fernandez-Miranda (2010) [50]	Wistar rat	EESBS	Surgeons	n/a
Valentine (2011, 2016, 2016) [51–53]	Sheep^c^	ICAI	Authors	n/a
Mladina (2013) [54]	Lamb	CSFL	Authors	n/a
Awad (2014) [55]	Sheep	ERT	Surgeons	6$
Skitarelić (2015) [56]	Lamb	EESS	Authors	2$
Isaacson (2015) [57]	Sheep	ETr	Authors	n/a
Mallmann (2016) [58]	Lamb	EESS	Surgeons	n/a
Mladina (2018) [49]	Lamb	ESSBS	Residents	3–4$

*$* US dollars, *CSFLR* cerebrospinal fluid leak repair, *Dev*. developers, *EESBS* endoscopic endonasal skull-base surgery, *EESS* endoscopic endonasal sinus surgery, *ERT* endoscopic rhinology tasks, *ETr* endscopic training, *FA* first author, *ICAI*R internal carotid artery injury repair, n/a not available, *YOP* year of publication

^c^This model is hybrid as the SIMONT artificial model is also used

### Artificial models

By definition, these models are fabricated artificially. They can be divided into two major categories: the first is represented by the so-called box-trainers (Table 3), while the second comprises anatomically more realistic models (Table 4). Artificial training models are considered the most cost-effective alternative to cadaver-based training [89]. Tables 3 and 4 report each model costs when available.

**Table 3:** Non-anatomical (box-trainer). The table reports the box-trainers, the simulated task, who assessed the model, and whenever available, the cost

FA/Dev. (YOP/nation)	Box-Trainer			Simulated Task	Assessment	Cost/price
	Type	T	A			
Hirayama (2013) [59]	E	-	-	HI	Surgeons	180$
Jusue-Torres (2013) [60]	F^d^	-	-	EEA	Surgeons	10$
Singh (2016) [61]	E	-	-	EES	Surgeons	100–150$
Sanromán-Álvarez (2017) [62]	EF	Egg	-	TP	Residents	n/a
Berkowitz (2017) [63]	E	-	-	TE	Students	n/a
Srivastav (2017) [64]	E^e^	-	-	HI	Novices	n/a
Xie (2018) [65]	EF	-	-	CSFLR	Surgeons	100$^f^
Altun (2020) [66]	F	CB	-	EETA	Experts	100$
Gallet (2021) [67]	EF	-	-	EES	Surgeons	n/a
Tikka (2022) [68]	F	-	-	EEA	Fellows	n/a
Bright (2022) [69]	E	-	-	TP	Surgeons	15$

*$* US dollars, *A* arachnoid, *CB* the tumor was simulated with the medullar content of a chicken bone, *CSFLR* cerebrospinal fluid leak repair, *Dev*. developers, *E* exercise based box-trainer, *EEA* endoscopic endonasal approach, *EES* endoscopic endonasal surgery, *EETA* endoscopic endonasal transsphenoidal approach, *EF* exercise and food based box-trainer, *Egg* chicken or quail egg used to mimic the content of the sella turcica, *F* food based box-trainer, *FA* first author, *HI* handling instruments, *n/a* not available, *T* tumor, *TE* transnasal endoscopy, *TP* transnasal procedures, *YOP* year of publication

^d^Additional training simulation with the chicken wing inside a Phacon training model

^e^Improved version of Sing et al. box-trainer

^f^15$ for the disposable parts

**Table 4:** Training models. The table reports the training model, including *m* models, *M* models, and EggHead, the simulated task, who assessed the model, and whenever available, the cost. The sub-column score gives an overall evaluation on the anatomical accuracy of the model; points are given according to the design of the model: +1 point per m models or +2 points for M; +1 or +2 points to the details reproduced such as the skin, the dura mother, the optic nerve, or the ICA; and +1 point if the tumor or the arachnoid are reproduced

FA/Dev. (YOP/nation)	Training model					Simulated task	Assessment	Cost/price
	Type/name	Anatomy	T	A	Score			
Briner (2007) [70]	m	PS	-	-	3/5	ESS	Experts	n/a
Chen (2010) [71]	M	Head	-	-	4/5	EETA	Authors	n/a
Okuda (2010) [72]	m	SB	Egg	-	2/5	EETA	Authors	n/a
Waran (2012) [73]	M	SB	-	-	3/5	ETr	Experts	n/a
Okuda (2014) [74]	M	Head	Egg	-	3/5	TP	Surgeons	n/a
Chan (2015) [75]	m	SB	-	-	2/5	ETr	Experts	600$^g^
Chan (2015) [75]	M	SB	-	-	3/5	ESS	Expert	900$^g^
Narayanan (2015) [76]	M	Head	-	-	3/5	TP	Surgeons	n/a
Engel (2015) [77]	M	Head	Egg	-	4/5	TP	Surgeons	2500^h^
Tai (2016) [78]	M	Head	-	-	3/5	EEA	Surgeons	500$^i^
Kashapov (2016) [79]	M	Head	SR	-	4/5	ESBS	Surgeons	200$^h^
Wen (2016) [80]	M	Head	Egg	-	4/5	EETA	Surgeons	n/a
Shah (2016) [81]	m	Head	-	-	2/5	EETA	Resident	n/a
Zheng (2018) [82]	M	Head	-	-	4/5	ESBS	Experts	500$^i^
Masuda (2018) [83]	M	Head	-	-	4/5	EETA	Authors	n/a
Lin (2018) [84]	M	SR	ns	ns^j^	4/5	ETA	Experts	n/a
Ding (2019) [85]	M	Head	Egg	-	5/5	EETA	Residents	n/a
Zheng (2019) [86]	M^k^	SR	ns	-	5/5	EETA	Residents	n/a
Shen (2020) [87]	M	Head	SG	-	5/5	EETA	Authors	n/a
London (2021) [88]	m	Head	-	-	3/5	PESS	Surgeons	63$^g^
Masalha (2021) [89]	M	Head	-	-	4/5	CSFLR	Surgeons	80$^g^
Lai (2021) [90]	M	Head	Pm	-	5/5	ESBS	Experts	570$^g^
Li (2022) [46]	m	Head	Egg	-	2/5	ETTA	Surgeons	200Y^g^
Li (2022) [46]	M	Head	-	-	4/5	ETTA	Surgeons	3000Y^g^
Pro Delphus (Brazil) [91]	SIMONT [92]	Head	ns	-		NT[93]	[51–53, 94, 95]	3798$–1630$
JMC (Japan) [96, 97]	Kezlex [98–100]	Head	-	-		NT	[101–104]	n/a

*$* US dollars, *€* Euros, *CSFLR* cerebrospinal fluid leak repair, *Dev*. developers, *EEA* endoscopic endonasal approach, *EETA* endoscopic endonasal transsphenoidal approach, Egg chicken or quail egg used to mimic the content of the sella turcica, *ESBS* endoscopic skull-base surgery, *ESS* endoscopic sinus surgery, *ETA* endoscopic transsphenoidal approach, *ETr* endoscopic training, *ETTA* endoscopic transnasal transsphenoidal approach, *FA* first author, *m* mono-material, *M* multi-material, *n/a* not available, *ns* not specified, *NT* neurosurgical training, *PESS* pediatric endoscopic sinus surgery, *Pm* polyvinyl alcohol cryogel mixed with water, *PS* paranasal sinus, *SB* skull-base, *SG* silica gel, *SR* sellar region, *SBS* skull base surgery, *T* tumor, *TP* transsphenoidal procedure, *Y* yuan, *YOP* year of publication

^g^Cost per model

^h^Production cost

^I^Material cost

^j^The arachnoid was reproduced for a non-transsphenoidal training model

^k^In the article are reported 3 different model; the M was chosen as it is considered the best option by the authors

### Box-trainer

If compared to CT-based models, the anatomical accuracy of box-trainers is significantly lower, but they are generally easier to fabricate and cheaper.

The models under this category are characterized by a box with two holes representing the head and the nostrils. Different materials and training modules can be used inside the box, e.g., chicken wing or tangerine [60, 62, 66, 68], rings, and pegs, to create specifically designed exercises [59, 61, 63–65, 69]. These models aim to develop the surgeon’s dexterity [68]. The box-trainers are reported in Table 3.

### CT-based models

These training models are developed from patient-specific CT data (Table 4). The overall level of anatomical accuracy is strongly related to the design, materials, and technology used.

Some are mono-material solutions [70, 72, 81], while others are multi-material to reproduce the different tissues of the human head more accurately [66, 71, 73, 74, 76–80, 82, 83, 85–88, 101, 102, 105].

A brilliant and cheap solution frequently incorporated in CT-based models is the “EggHead,” described by Engel et al. [77]: a chicken or quail egg reproduces the sellar region anatomy [46, 67, 72, 74, 77, 80, 85]. The eggshell mimics the sphenoid bone, the vitelline membrane is the dura mater, and the albumen and yolk represent the contents of the sella. According to Wen et al. [80], the egg may be raw or soft-boiled.

Among multi-material training models, some are commercially available, such as SIMONT by ProDelphus [91], Kezlex: A22 [98], A39 [99], and A43 [100] by Japan Medical Company [96]. The Sinus Model Otorhino Neuro Trainer, SIMONT - Otorhino Surgical Trainer, is the training model developed which allows the performing of many neurosurgical operations [93], including removing the pituitary adenoma. One of the most innovative features is Neoderma®, the material developed by Pro Delphus used to mimic the mechanical properties of the skin and the mucous membranes [94, 95, 104]. The model is available on the website [92] for US$ 3798.00, while the portable version costs US$ 1630. In the literature, its use has been described by Valentine et al. [51–53].

Kezlex is a series of training models developed by Japan Medical Company [96]. Among all the training solutions [97], the most pertinent are models A22 [98], A39 [99], and A43 [100]. Oyama et al. described their experience with the A22 for various neurosurgical approaches. Maza et al. [101] described the A43 model. This training model was developed to help the neurosurgeon deal with a catastrophic ICA injury. The cost is not reported on the website, but Muto et al. [102] reported in their article the cost of the A43 model of $4000 plus $250 for the reusable platform.

### Simulators

Training simulators can be divided into two categories, as they are either based on virtual (VR) or augmented reality (AR). Table 5 reports the relative costs of each model when available.

**Table 5:** Training simulators. The table reports VR and AR simulators. In the sub-column, “Devices” is reported the tools that surgeons use to interact with the simulator, while the sub-column “Model” is for the AR simulators only, and it is reported the physical model where the simulation is performed

FA/Dev. (YOP/nation)	Training simulator					Simulated task	Assessment	Cost
	Type/name	Devices	Model	T	A			
Wolfsberger (2004, 2006) [106, 107]	VR	Joystick	-	T	-	EETA	Authors	n/a
Pöβneck (2005) [108]	VR	Haptic	-	-	-	ESS	Residents	n/a
Neubauer (2005) [109]	VR	Joystick	-	T	-	EETA	Authors	n/a
Dixon (2011, 2012, 2014) [110–112]	AR	Instruments	CH	-	-	ESBS	Experts	n/a
Prisman (2011) [113]	AR	Instruments	CH	-	-	ESBS	Surgeons	n/a
De Notaris (2013) [114]	VR	Instruments	CH	-	-	EEA	Residents	n/a
Varshney (2014) [115]	VR^l^ [116]	Haptic	-	-	-	ESS	Authors	n/a
Li (2016) [117]	AR	Instruments	m, CH	-	-	ESBS	Surgeons	n/a
Won (2018) [118]	VR [119]	Haptic	-	-	-	ESBS	Authors	n/a
Barber (2018) [120]	AR	Instruments	m	T	-	ESS	Authors	1000$
Heredia-Pérez (2019) [121]	VR	Haptic	-	T	-	REETA	Surgeons	n/a
Lai (2020) [122, 123]	AR	Instruments	m	-	-	ESBS	-	n/a
Kim (2020) [124]	VR	Haptic	-	-	-	ESSBS	Experts	n/a
Cai (2022) [125]	AR^m^	-	-	T	-	PTR	-	n/a
UKE (Germany) [126, 127]	Voxel-Man Sinus	Instruments	-	-	-	ESS [128]	[129]	145,255.95$ [130, 131]
NRC and NeuroSim (Canada) [116]	NeuroVR	Instruments	-	T	-	NT [132]	[115, 133]	n/a [134]
Phacon GmbH (Germany) [135]	Phacon	Instruments	M	-	-	NT [136, 137]	[60, 138]	1870€ [139]; 8910€ [140]; 290€ [141]
UpSurgeOn S.r.l. (Italy) [142]	TNS	Instruments	M	T	-	EETA [143]	[144]	599–€699€ [145]; disposable n/a [146]

*$* US dollars, *€* Euros, *A* arachnoid, *CH* cadaver head, *Dev*. developers, disposable n/a disposable cavities cost not available, *EEA* endoscopic endonasal approach, *EETA* endoscopic endonasal transsphenoidal approach, *ESBS* endoscopic skull-base surgery, *ESS* endoscopic sinus surgery, *ESSBS* endoscopic sinus and skull-base surgery, *FA* first author, *m* mono-material head, *M* multi-material head, *n/a* not available, *NT* neurosurgical training, *disp* disposable cavities, *PTR* pituitary tumor resection, *RTBTR* robotic transsphenoidal brain tumor resection, *YOP* year of publication

^l^Simulator developed upon the NeuroVR platform

^m^Application for AR training simulator

### Virtual reality

VR simulators consist of a PC with a virtual environment software that represents the patient’s data, and the surgeons interact with it by simulating actual surgeries using joysticks [106, 107, 109], special haptic devices [108, 115, 118, 121, 147], or surgical instruments [114]. Virtual reality simulators are a technologically advanced alternative to train surgeons to perform complex surgeries before they enter the operating room [3, 60].

Two different VR simulators were found to be available online, the NeuroVR and Voxel-Man Sinus.

The NeuroTouch-Endo, now NeuroVR, is the training simulator developed by NRC and NeuroSim [116] (Canada); it is a VR simulator that simulates endoscopic transnasal procedures [132] with MRI data for patient-specific features. In addition, it has haptic devices that provide force feedback [115, 133], and it is available on the CAE website [134].

The Voxel-Man Sinus [126] is the training simulator developed by the University Medical Center Hamburg-Eppendorf (Germany) [127] for paranasal sinus surgery [128]. The Voxel-Man provides an accurate haptic and visual representation of surgery and is based on standard PC hardware [129]. The Voxel-Man can be purchased [130] for $ 145,255.95$ [131].

### Augmented reality simulators

AR simulators are VR simulators where surgeons interact with a physical, CT-based [75, 117, 120, 122, 123], or cadaver [110–114, 117] head. Additionally, Cai et al. [125] developed an application that can be used for AR simulators. Two models were available for purchase, the Phacon Sinus Trainer and the TNS Box.

The PHACON Sinus Trainer comprises a series of simulators developed by Phacon GmbH (Germany) [135]. The most suitable for this review were found with the web research: the [S-00005] PHACON Sinus Trainer [137], available at 8.910€ [140], and the [S-00007] PHACON Sinus Assistant [136], purchasable at 1.870€ [139]. The module for the transnasal approach, the [SN-ah] PHACON Sinus Patient “Meyer” – pituitary tumor, can be purchased separately for 290€ [141]. The simulator consists of a multi-material modular head connected via visual registration to specially developed software that assists the neurosurgeon by providing CT data displayed as a virtual 3D model; it can automatically detect injuries to high-risk structures.

The TNS Box is one of the multiple simulators developed by UpSurgeOn [142]. It consists of an anatomically accurate modular and multi-material simulator designed explicitly for the transsphenoidal approach to the pituitary gland. The simulator comprises an external box with a disposable nasal cavity and a face mask on the front. The TNS is provided with an App available on the App Store or Google Play, which improves the training experience with a virtual reality environment [143]. The TNS is now available at UpSurgeOn website [145] at €599–€699. It is also possible to purchase disposable nasal cavities separately [146]. Two articles reported a positive experience with the simulators [144, 148].

### Models and simulators for surgical planning

Table 6 reports models and simulators conceived for surgical planning, which are not included in this systematic review but might help neurosurgeons improve their knowledge on the subject [28, 149–154, 156–158].

**Table 6:** Surgical planning training models/simulators: the model and simulators reported in this table are those designed to help neurosurgeon during the preoperative planning. BR Blue Resin (Vero Cyan®, Stratasys)

FA/Dev. (YOP/nation)	Surgical planning model/simulator				Planning	Assessment	Cost
	Type/name	Anatomy	T	A			
Shinomiya (2018) [28]	m	SR	BR	-	EETA	Experts	n/a
Zhang et al. (2018) [149]	m	PS-SB	-	-	PSS	Experts	3$/m
Huang (2019, 2021) [150, 151]	m	SR	T	-	EETA	Authors	900–1500Y
Panesar (2021) [152]	M	SB	T	-	ESBS	Experts	3–40$/m
Chaudhary (2021) [153]	m	SR	T	-	EETA	Experts	n/a
Chopra (2021) [154]	m	SR	T	-	EETA	Authors	n/a
Bracco Group (Italy) [155]	Dextroscope	Head	-	-	NT	[156–158]	n/a

*$* US dollars, *A* arachnoid, *BR* blue resin (Vero Cyan®, Stratasys), *Dev.* developers, *EETA* endoscopic endonasal transsphenoidal approach, *ESBS* endoscopic skull-base surgery, *FA* first author, *m* mono-material, M multi-material, *n/a* not available, *NT* neurosurgical training, *PSS* paranasal sinus surgery, *PS-SB* paranasal sinus–skull-base, *SB* skull-base, *SR* sellar region, *T* tumor, *Y* yuan, *YOP* year of publication

## Discussion

It is recognized that the endoscopic endonasal transsphenoidal approach has a long learning curve [6]. To ensure safe and effective surgery, it is crucial to have excellent hand-eye coordination under the endoscopic vision and make sound clinical and intraoperative judgments. The required confidence can only be achieved after many surgeries in the operating room. However, this learning process can be sped up with proper training in a safe environment outside the operating room.

This systematic review aimed to show all alternatives for training in endoscopic transsphenoidal surgery. We found four categories of training systems: enhanced cadaver head training models, animal models, training models (CT-based, box-trainer, and EggHead), and training simulators (virtual reality and augmented reality).

Human cadaver heads remain the gold standard for training: the anatomical reliability is still higher if compared to every other option [21]. However, their low availability [18, 40, 45] and the fact that they are suitable for limited training experience make them an expensive and not easily accessible option [42]: the cost of one human cadaveric head ranges from almost 600$ [48] to 1000$ [79], while Mladina et al. [49] reported a cost of 1520$ per resident for training. In addition, the maintenance costs of anatomy laboratories are high [17]. Using animal heads is a cheap and readily available option, but the anatomy is divergent [56]. Nevertheless, they can be considered a good alternative as an inexpensive and simple system to teach residents the dexterity required to fully exploit the more expensive cadaver head, as stated by Mladina et al. [49]. Their main advantages are the costs which are lower than 6$ [49, 55, 56], making them the cheapest solution for initial training.

Compared to human and animal specimens, training models have the advantage of being versatile. The developers can choose the anatomical accuracy level they want to obtain, which is directly related to the costs of the system. Modular solutions, in which not all parts are disposable, are a way to optimize the costs of this solution.

Among the different solutions, the box-trainers are cheaper and easier to fabricate. However, the low degree of anatomical accuracy makes them suitable as a first tool to teach how to handle surgical instruments in the narrow space of the nasal cavities, and they can be a useful first experience before training with more expensive models like the cadaveric head [68].

CT-based training models, on the other hand, potentially have a significantly higher level of accuracy related to the design complexity and the background knowledge required. The EggHead represents a brilliant solution as it mimics the sellar region with a chicken or quail egg in an economical and repeatable way [46, 72, 74, 77, 80, 85]. What needs to be added is a reproduction of blood and CSF [76]; the latter was implemented only in the training model of Mashala et al. [89]. Costs are generally low but cannot be compared to each other due to the different criteria by which they were determined by the authors, as reported in Table 4. They can be divided into three categories: cost per model, material cost, and production cost.

VR training simulators provide visually the most complete experience to neurosurgeons. Their main advantage is the fact that the simulation can ideally be repeated an infinite number of times [17]. In addition, some of them also have a real-time feedback system that provides information about the position of the instruments, the level of forces reached, and the performances of the trainees [18, 115, 129, 133]. However, the lack of a “physical head” where to perform the surgery can be limiting, even if many sensors and haptic devices have been studied and added [121]. Another defect of some VR systems is the low quality of the visual effects and the fact that the instruments used during training sessions differ from those used in the operating room [106–109, 116, 118, 121, 124, 131]. The initial costs of VR training simulators are the highest among the different solutions; i.e., the Voxel-Man Sinus training simulator is available for 145,255.95$ [130, 131]. However, the fact that surgeries can be simulated an indefinite number of times makes the cost of a single training session low if the system is used frequently.

AR simulators with cadaver heads may be the best solution for residents as they provide the best anatomy from the cadaver head and real-time feedback from the VR environment [110–112, 117, 156]. However, they may also be the most expensive solutions: a better trade-off to reduce cost may be an AR simulator based on a multi-material head. The costs available for the training simulators are those of the Phacon, 8910€ [140], or 1870€ [139] and 290€ [141] for the cartridge, and those of the TNS, 599–699€ [145], plus the costs of the disposable cartridge, which it is not reported. Similar to CT-based training models, developing VR and AR simulators requires a high level of knowledge.

Finally, this review documents what is missing in most training solutions. Most are dedicated to the phase of the approach in surgery, while only a minority have developed simulators for sellar tumors and suprasellar arachnoid. Except for VR simulators, where the pituitary adenoma was implemented virtually, the sellar tumor has been simulated only in a few models using different materials. In addition, ECH models have been modified to allow training for dealing with ICA intra-operative rupture and CSF leak. We believe it might be of interest to develop a modular training model that provides a realistic simulation of both sellar tumors and suprasellar arachnoid to provide a cost-efficient way to train future generations not only in the surgical approach but also in the management of sellar tumors of different consistencies and the preservation of the arachnoid.

## Limits of the study

The limit of this systematic review could be the lack of some data of the training models/simulators (e.g., the cost of the training model) and therefore the difficulty of comparing the models. Furthermore, not all models that are being developed are available at the moment. We expect that further improvements will be made soon in the field.

## Conclusions

The training solutions for endoscopic transsphenoidal surgery are cadaveric (human or animal) or artificial models and virtual reality simulators. Human cadaveric specimens constitute the gold standard, as they provide a realistic environment, which specific modifications for managing ICA rupture, CSF leak, and tumor removal can enhance. Their availability is though relatively low due to relatively high costs. Virtual reality simulators and artificial models provided an excellent alternative. However, the lack of haptic realism and anatomical fidelity makes them ideal for learning the basics. Augment reality applied to cadaver-based models is an exciting solution that might be further developed in the near future.

Most artificial models do not provide a realistic and cost-efficient simulation of the most delicate and relatively common phase of surgery, i.e., tumor removal with arachnoid preservation; current research should optimize this to train future neurosurgical generations efficiently and safely.

## Data Availability

This review was performed by searching articles on Pubmed and Scopus.
